# Neurodevelopmental Crossroads: The Role of Masked Autism Spectrum Disorder in the Development of Persecutory Beliefs Within Forensic Psychiatry

**DOI:** 10.1192/bjo.2026.11869

**Published:** 2026-06-30

**Authors:** Anushka Musharrat

**Affiliations:** Essex Partnership University NHS foundation TRUST, Essex, United Kingdom

## Abstract

**Aims:**

This report details the forensic admission of Ms X, a woman in her 40s, following an index offence of Grievous Bodily Harm (GBH) driven by fixed persecutory beliefs. It underscores the clinical challenge of identifying Autism Spectrum Disorder (ASD) when masked by “social camouflaging” or obscured by somatic comorbidities. Historically, Ms X’s difficulties were attributed to Chronic Fatigue Syndrome and depression. At the same time, her forensic presentation was initially diagnosed as “Delusional Disorder,” illustrating the risks of diagnostic overshadowing in forensic risk assessment.

**Methods:**

Ms X presented with persistent beliefs that an online group had compromised her devices and that neighbours were surreptitiously filming her. These beliefs culminated in her confronting neighbours while armed. On admission, her presentation diverged from primary psychosis; she exhibited limited conversational reciprocity, sensory hypersensitivities (noise and olfaction), and a narrow, obsessive preoccupation with cyber-security. A retrospective developmental history revealed core ASD traits missed due to female-typical compensation strategies, including long-standing social aversion and ritualistic behaviours. This led to a formal diagnosis of ASD, replacing the previous diagnosis of primary Delusional Disorder.

**Results:**

The clinical formulation reframed Ms X’s “delusions” as intense manifestations of autistic cognitive style. Her beliefs were formed through a concrete, literal interpretation of ambiguous stimuli; for example, interpreting the online phrase “I see you” as evidence of physical surveillance. Her inherent cognitive rigidity rendered these beliefs resistant to challenge. Furthermore, deficits in Theory of Mind caused her to misattribute neutral or ordinary environmental actions as intentional threats. Consequently, the index offence was formulated as a reactive defence rooted in neurodevelopmental rigidity and emotional dysregulation, rather than a psychotic break.

**Conclusion:**

This case highlights the imperative for forensic practitioners to screen for masked ASD in women, particularly when intense somatic or mood symptoms are present. Understanding ASD-related rigidity is crucial for accurate risk formulation and legal mitigation. Management must shift from traditional antipsychotic protocols toward integrated, specialised psycho-social interventions–such as Neurodevelopmental Outreach Services–focused on building ASD-specific insight and adaptive coping mechanisms.

